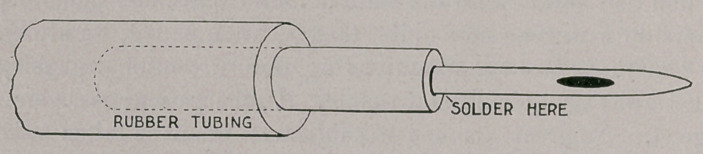# A Case of Milk-Fever

**Published:** 1903-05

**Authors:** E. H. Lehnert

**Affiliations:** Storrs, Conn.


### REPORTS OF CASES.

### A CASE OF MILK-FEVER. A NEW TREATMENT.

By E. H. Lehnert, D.V.S.,

STORRS, CONN.

As a usual thing we do not attach a great deal of importance to the results obtained and records made in the treatment of a single case, but in this particular instance I feel justified in reporting in detail a case of parturient apoplexy which has just been discharged, perfectly cured and under treatment for only four days. Some of my readers will undoubtedly say, "Strong language when speaking of milk-fever." Well, perhaps so, but please suspend judgment until you have carefully read the article.

The writer has had some considerable experience with this disease, and had gotten into the state of mind that most general practitioners do with reference to it—that is, when called, we always try to do something besides administering the conventional physic, etc., and get away as quickly as possible. If the patient recovers, well and good. No great amount of subjective credit is taken as to the curative value of any therapeutic agent employed; in fact, it is a case that one would rather not have been called to attend.

The Schmidt treatment has been as unsatisfactory (to the writer, at least) as has been any and every other remedy employed. When, however, an article in a certain breeder's publication on the use of oxygen gas injected into the udder was discovered (for it had to be discovered, as it was given no conspicuous place whatever), it seemed to me that there was a remedy reasonable and scientific and well worthy of a trial, the only difficulty being the tank of oxygen under pressure; this we had on hand—a tank used in connection with a calcium-light stereopticon. The apparatus used for injecting the gas into the udder consisted of an ordinary milking tube, to the bottom or base of which was soldered a tube sufficiently large to admit of the attachment of the rubber tubing. See illustration, page 308.

The administration is not at all difficult. First, milk out the udder, then disinfect teats and tube; next insert the tube and allow the gas to flow in gently until quarter is filled; then compress end of teat and work the gas thoroughly into the gland tissue by massage. There seems to be no deleterious effect from the gas in the udder, no inflammation, and no suppression of the milk other than you generally get in these cases.

The writer was called to this case at about 5 P.M. April 8th. When seen the animal was down, head on one side and presenting every symptom of the insensible stage of this disease, breathing heavily, with occasional convulsive movements of the limbs, somewhat bloated and very little reflex noticeable in touching the eyeball. Cow had calved the day before and had been down for several hours. The article on oxygen treatment being prominent in my mind, I advised the owner to allow the cow to be taken to the college for treatment, and after being informed that she would probably die in any event, he agreed to my proposition. At about 7 P.M. she was loaded upon an ordinary farm wagon and carried nearly three miles in a cold driving rain, arriving at the college at about 8 P.M. At that time she showed marked symptoms of approaching death—eyes set and devoid of reflex, gasping for breath, lying flat on one side with tympanites somewhat more marked. Soon after beginning the injection of the oxygen it became very evident that she would die unless something was done immediately; although we did not expect that it would prove of any benefit, she was tapped, however; her breathing soon came easier, and the injection of the gas was continued after an interval of about thirty minutes. (Delay in fitting tube.) In one hour's time, 9 P. M., eyes were closed and upon stimulation showed evident reflex movement, there were also nervous tremblings or twitchings of the limbs. Temperature, 101°.

At 9.30 P.M. she was lying quietly flat on side, breathing easily and regularly, without any throat sounds whatever.

At 11.30 P.M. she was lying in a natural position, that is, on the sternum, looking bright and taking an evident interest in all that transpired.

At 12.30 A.M., April 9, lying natural. Injection repeated.

At 6 A.M. she was upon her feet moving about and looking for something to eat. Temperature, 101.3°; pulse, 60. Injection repeated after milking out the udder. She was given a small feed of hay and a sloppy bran-mash, to which she immediately turned her attention. A little later she was given a quart of raw oil, although shortly afterward it was seen that she did not need it, as her bowels were in perfect condition

At 12 noon, temperature, 101.2° ; pulse, 60. Had eaten a little of the hay and mash. Udder milked out and injection repeated.

At 7 P.M. Temperature, 101° ; pulse, 60. Apparently feeling about normal.

At 8 A.M., April 10th, temperature, 101° ; pulse, 54. General improvement marked.

At 12 noon, temperature, 101.2° ; pulse, 60.

At 6 P.M. Temperature, 101.3° ; pulse, 60.

At 6 A.M., April 11th. Temperature, 101.2° ; pulse, 57. Lying naturally, chewing cud.

At 12 noon, temperature, 101.1° ; pulse, 56.

At 6 P.M. Temperature, 101.2° ; pulse, 56.

At 9 A.M., April 12th. Temperature, 100.3° ; pulse, 56. Gave seven quarts of milk.

At 12 noon, temperature, 101° ; pulse, 56. As the day was warm she was tied out to eat grass for an hour. At 6 P.M. she gave about four quarts of milk.

It is not supposed that the profession will accept this record as final in the treatment of this disease, but it is hoped that it will stimulate them and wake them up to the fact that here is a field for investigation with a path outlined. By reporting their several experiences to our magazines we shall in the end arrive at something definite. This outcome we all shall look forward to and welcome with almost if not quite as much enthusiasm as the advent of serum therapy or even tuberculin.

The article to which reference has been made stated that M. Knusel, a veterinarian in Lucerne, Switzerland, had treated 22 cases in this manner, all of which recovered, and in general the recovery was rapid and marked, as in the case here reported—although in most instances more so. For example, the statement was made that they were up and looking for food in from thirty to sixty minutes after a single injection of the gas. It is offered by way of explanation of the action of oxygen in this disease, that in all probability the germ is anaërobic and, therefore, is destroyed or, at least, rendered inactive by the presence of the gas in the circulation. This theory is borne out by the fact that relapses may occur, but relief is immediately secured by a fresh injection of oxygen.

Veterinarians, this is no "fake." Give it a trial and report your cases to the JOURNAL OF COMPARATIVE MEDICINE, etc. I can offer no definite figures, but it seems to me that at a moderate expense a five-gallon tank might be secured, having a wide diameter and setting low so as not to be unwieldy.

In all probability we shall have further reports to make along this line, as the college has undertaken to treat cases free of charge in order that a more or less exhaustive series of experiments may be carried out.

All letters of inquiry addressed to the writer will be cheerfully answered.

## Figures and Tables

**Figure f1:**